# *N*,*N*′-Dibenzyl-*N*′′-(2,4-difluoro­benzo­yl)-*N*,*N*′-dimethyl­phospho­ric triamide

**DOI:** 10.1107/S1600536811055115

**Published:** 2012-01-07

**Authors:** Mehrdad Pourayoubi, Samad Shoghpour, Laura Torre-Fernández, Santiago García-Granda

**Affiliations:** a Department of Chemistry, Ferdowsi University of Mashhad, Mashhad 91779, Iran; b Departamento de Química Física y Analítica, Facultad de Química, Universidad de Oviedo – CINN, C/ Julián Clavería, 8, 33006 Oviedo, Asturias, Spain

## Abstract

In the title mol­ecule, C_23_H_24_F_2_N_3_O_2_P, the P atom is in a distorted tetra­hedral P(=O)(N)(N)_2_ environment, with the bond angles around the P atom in the range 106.78 (11)–114.10 (13)°. The phosphoryl and carbonyl groups, which are separated by an N atom, adopt an *anti* orientation relative to each other. In the C(=O)NHP(=O) fragment, the P—N bond is longer [1.683 (2) Å] and the O—P—N angle is smaller [106.78 (11)°] than the other P—N bonds [1.613 (2) and 1.632 (2) Å] and O—P—N bond angles [114.10 (13) and 110.83 (12)°], respectively. The N atoms have *sp* ^2^ character. In the crystal, pairs of P=O⋯H—N hydrogen bonds form inversion dimers with *R*_2_^2^(8) ring motifs.

## Related literature

For hydrogen-bond patterns in compounds with formula *R*C(O)NHP(O)[N*R* ^1^ *R* ^2^]_2_ and *R*C(O)NHP(O)[NH*R* ^1^]_2_ and for the discussion of different C(=O) *versus* P(=O) orientations in the C(O)NHP(O) fragment, see: Toghraee *et al.* (2011 ▶). For hydrogen-bond strengths in cyclic hydrogen-bond motifs and for bond lengths and angles, see: Pourayoubi *et al.* (2011 ▶). For graph-set analysis of hydrogen-bonds motifs, see: Bernstein *et al.* (1995 ▶). For the synthesis of the starting phospho­rous–chlorine compound, see: Pourayoubi *et al.* (2010 ▶).
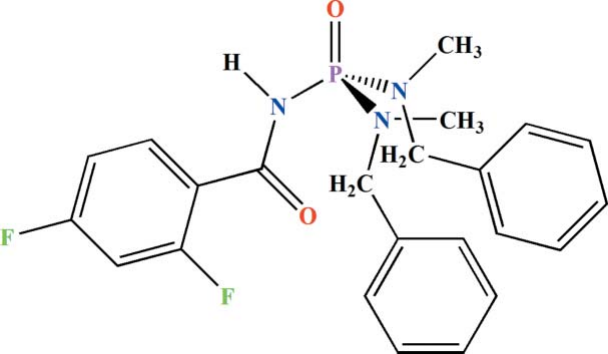


## Experimental

### 

#### Crystal data

C_23_H_24_F_2_N_3_O_2_P
*M* *_r_* = 443.42
Triclinic, 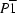

*a* = 10.3619 (6) Å
*b* = 10.7721 (10) Å
*c* = 11.6433 (8) Å
α = 70.523 (7)°
β = 72.495 (5)°
γ = 70.197 (7)°
*V* = 1126.65 (15) Å^3^
*Z* = 2
Cu *K*α radiation
μ = 1.44 mm^−1^
*T* = 300 K
0.17 × 0.14 × 0.03 mm

#### Data collection

Agilent Xcalibur Gemini R diffractometer
Absorption correction: multi-scan (*CrysAlis PRO*; Agilent, 2011 ▶) *T*_min_ = 0.954, *T*_max_ = 1.000
10022 measured reflections
4209 independent reflections
2758 reflections with *I* > 2σ(*I*)
*R*_int_ = 0.044

#### Refinement

*R*[*F* ^2^ > 2σ(*F* ^2^)] = 0.051
*wR*(*F* ^2^) = 0.141
*S* = 1.01
4209 reflections
268 parameters
H-atom parameters constrained
Δρ_max_ = 0.47 e Å^−3^
Δρ_min_ = −0.27 e Å^−3^

### 

Data collection: *CrysAlis PRO* (Agilent, 2011 ▶); cell refinement: *CrysAlis PRO*; data reduction: *CrysAlis PRO*; program(s) used to solve structure: *SIR92* (Altomare *et al.*, 1994 ▶); program(s) used to refine structure: *SHELXL97* (Sheldrick, 2008 ▶); molecular graphics: *Mercury* (Macrae *et al.*, 2008 ▶); software used to prepare material for publication: *WinGX* (Farrugia, 1999 ▶) and *enCIFer* (Allen *et al.*, 2004 ▶).

## Supplementary Material

Crystal structure: contains datablock(s) I, global. DOI: 10.1107/S1600536811055115/fy2035sup1.cif

Structure factors: contains datablock(s) I. DOI: 10.1107/S1600536811055115/fy2035Isup2.hkl

Additional supplementary materials:  crystallographic information; 3D view; checkCIF report

## Figures and Tables

**Table 1:** Hydrogen-bond geometry (Å, °)

*D*—H⋯*A*	*D*—H	H⋯*A*	*D*⋯*A*	*D*—H⋯*A*
N2—H2⋯O5^i^	0.86	1.96	2.812 (3)	171

Symmetry code: (i) 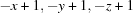.

**Fig. 1.**
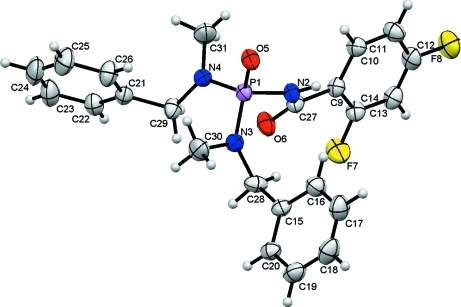
The molecular structure of the title compound with ellipsoids shown at the 30% probability level.

**Fig. 2.**
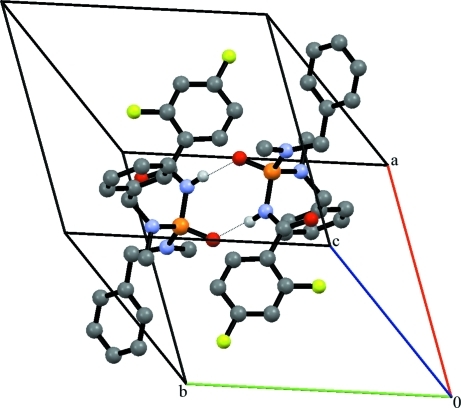
A view of the centrosymmetric dimer formed by H-bonding. The H atoms not involved in the hydrogen bonding interaction have been omitted for clarity.

## supplementary crystallographic information

### Comment

In a recently published paper, the collective behavior of hydrogen bonds (HBs) in the crystal packing of phosphoramidates having a C(═O)NHP(═O)(N)_2_ and C(═O)NHP(═O)(NH)_2_ skeletons were considered (Toghraee *et al.*, 2011). The authors attempted to arrive at some empirical rules which are useful for predicting hydrogen-bond patterns in systems having "two H-acceptors and one H-donor site" and "two H-acceptors and three H-donor sites". As a continuation, the hydrogen bond strengths in such systems were analyzed based on hydrogen bond motifs (Pourayoubi *et al.*, 2011).

The structure determination of the title molecule, P(O)[NHC(O)C_6_H_3_(2,4-F_2_)][N(CH_3_)(CH_2_C_6_H_5_)]_2_ (Fig. 1), was performed according to our interest in the collection of structural data related to new compounds having a C(O)NHP(O) skeleton.

The phosphoryl and the carbonyl groups in the title molecule adopt an *anti* orientation relative to each other similarly to most of the carbacylamidophosphates. However, a few examples with a *gauche* orientation of these two groups were also reported (Toghraee *et al.*, 2011). The phosphorus atom has a distorted tetrahedral configuration and the P═O, C═O, P—N bond lengths and P—N—C bond angles are within the expected values. In the crystal structure, pairs of intermolecular P═ O···H—N hydrogen bonds (Table 1) form centrosymmetric dimers, see Figure 2, as *R*_2_^2^(8) rings (Bernstein *et al.*, 1995).

### Experimental

Synthesis of 2,4-F_2_–C_6_H_3_C(O)NHP(O)Cl_2_. 2,4-F_2_–C_6_H_3_C(O)NHP(O)Cl_2_ was prepared similarly to the procedure which was used for the preparation of 2,6-F_2_–C_6_H_3_C(O)NHP(O)Cl_2_ (Pourayoubi *et al.*, 2010), but by using 2,4–F_2_–C_6_H_3_C(O)NH_2_ instead of 2,6–F_2_–C_6_H_3_C(O)NH_2_.

Synthesis of title molecule. To a solution of 2,4-F_2_–C_6_H_3_C(O)NHP(O)Cl_2_ (2 mmol) in CHCl_3_ (20 ml), a solution of *N*-methylbenzylamine (8 mmol) in CHCl_3_ (5 ml) was added dropwise at 273 K. After 4 h stirring, the solvent was evaporated in vacuum and then the resulting solid was washed with distilled water. Single crystals of title compound were obtained from a mixture of CHCl_3_ and n-C_7_H_16_ (5 to 1 v/v) after slow evaporation at room temperature. IR (KBr, cm^-1^): 3064, 3043, 2872, 1683 (C═O), 1615, 1453, 1343, 1265, 1215, 1175, 1155, 1128, 1088, 1017, 959, 877, 820, 791, 738, 714.

### Refinement

All H atoms were placed in geometrically calculated positions with N—H = 0.86 Å and C—H = 0.93 (aromatic C—H), 0.96 (CH_3_) or 0.97 Å (CH_2_). H atoms were refined in riding mode with U_iso_(H) = 1.5 U_eq_(C) for methyl groups and U_iso_(H) = 1.2 U_eq_(C/N) for all other H atoms.

### Crystal data

**Table:**

C_23_H_24_F_2_N_3_O_2_P	*Z* = 2
*M**_r_* = 443.42	*F*(000) = 464
Triclinic, *P*1	*D*_x_ = 1.307 Mg m^−^^3^
Hall symbol: -P 1	Cu *K*α radiation, λ = 1.54184 Å
*a* = 10.3619 (6) Å	Cell parameters from 2755 reflections
*b* = 10.7721 (10) Å	θ = 4.1–70.6°
*c* = 11.6433 (8) Å	µ = 1.44 mm^−^^1^
α = 70.523 (7)°	*T* = 300 K
β = 72.495 (5)°	Plate, colorless
γ = 70.197 (7)°	0.17 × 0.14 × 0.03 mm
*V* = 1126.65 (15) Å^3^	

### Data collection

**Table:**

Agilent Xcalibur Gemini R diffractometer	4209 independent reflections
Radiation source: Enhance (Cu) X-ray Source	2758 reflections with *I* > 2σ(*I*)
graphite	*R*_int_ = 0.044
Detector resolution: 10.2673 pixels mm^-1^	θ_max_ = 70.7°, θ_min_ = 4.1°
ω scans	*h* = −8→12
Absorption correction: multi-scan (CrysAlis PRO; Agilent, 2011)	*k* = −12→13
*T*_min_ = 0.954, *T*_max_ = 1.000	*l* = −14→14
10022 measured reflections	

### Refinement

**Table:**

Refinement on *F*^2^	Primary atom site location: structure-invariant direct methods
Least-squares matrix: full	Secondary atom site location: difference Fourier map
*R*[*F*^2^ > 2σ(*F*^2^)] = 0.051	Hydrogen site location: inferred from neighbouring sites
*wR*(*F*^2^) = 0.141	H-atom parameters constrained
*S* = 1.01	*w* = 1/[σ^2^(*F*_o_^2^) + (0.0585*P*)^2^] where *P* = (*F*_o_^2^ + 2*F*_c_^2^)/3
4209 reflections	(Δ/σ)_max_ < 0.001
268 parameters	Δρ_max_ = 0.47 e Å^−^^3^
0 restraints	Δρ_min_ = −0.27 e Å^−^^3^

### Special details

**Table:**

Geometry. All e.s.d.'s (except the e.s.d. in the dihedral angle between two l.s. planes) are estimated using the full covariance matrix. The cell e.s.d.'s are taken into account individually in the estimation of e.s.d.'s in distances, angles and torsion angles; correlations between e.s.d.'s in cell parameters are only used when they are defined by crystal symmetry. An approximate (isotropic) treatment of cell e.s.d.'s is used for estimating e.s.d.'s involving l.s. planes.
Refinement. Refinement of *F*^2^ against ALL reflections. The weighted *R*-factor *wR* and goodness of fit *S* are based on *F*^2^, conventional *R*-factors *R* are based on *F*, with *F* set to zero for negative *F*^2^. The threshold expression of *F*^2^ > σ(*F*^2^) is used only for calculating *R*-factors(gt) *etc*. and is not relevant to the choice of reflections for refinement. *R*-factors based on *F*^2^ are statistically about twice as large as those based on *F*, and *R*- factors based on ALL data will be even larger.

### Fractional atomic coordinates and isotropic or equivalent isotropic displacement parameters (Å^2^)

**Table:**

	*x*	*y*	*z*	*U*_iso_*/*U*_eq_	
P1	0.58022 (6)	0.27216 (7)	0.57829 (7)	0.0460 (2)	
N2	0.4103 (2)	0.3411 (2)	0.5698 (2)	0.0484 (5)	
H2	0.3866	0.4255	0.5297	0.058*	
N3	0.6579 (2)	0.1967 (3)	0.4676 (2)	0.0586 (6)	
N4	0.5904 (2)	0.1570 (2)	0.7108 (2)	0.0528 (6)	
O5	0.63610 (18)	0.38484 (19)	0.5704 (2)	0.0595 (5)	
O6	0.3324 (2)	0.1512 (2)	0.6516 (2)	0.0684 (6)	
F7	0.0748 (2)	0.2082 (2)	0.5985 (2)	0.0946 (7)	
F8	−0.24562 (18)	0.6018 (2)	0.6907 (2)	0.0991 (7)	
C9	0.1600 (3)	0.3630 (3)	0.6399 (3)	0.0503 (7)	
C10	0.1264 (3)	0.4859 (3)	0.6711 (3)	0.0594 (7)	
H10	0.1977	0.5144	0.6792	0.071*	
C11	−0.0098 (3)	0.5667 (4)	0.6902 (3)	0.0681 (6)	
H11	−0.0315	0.6484	0.7115	0.082*	
C12	−0.1121 (3)	0.5211 (4)	0.6763 (3)	0.0681 (6)	
C13	−0.0871 (3)	0.4028 (3)	0.6477 (3)	0.0604 (5)	
H13	−0.1592	0.3749	0.6400	0.072*	
C14	0.0492 (3)	0.3252 (3)	0.6305 (3)	0.0604 (5)	
C15	0.6261 (3)	0.2312 (3)	0.2566 (3)	0.0548 (7)	
C16	0.5939 (3)	0.3719 (3)	0.2161 (3)	0.0689 (9)	
H16	0.5505	0.4238	0.2739	0.083*	
C17	0.6249 (4)	0.4357 (4)	0.0925 (4)	0.0835 (11)	
H17	0.6024	0.5305	0.0666	0.100*	
C18	0.6890 (4)	0.3607 (5)	0.0060 (4)	0.0868 (12)	
H18	0.7097	0.4047	−0.0783	0.104*	
C19	0.7225 (3)	0.2209 (4)	0.0442 (3)	0.0767 (10)	
H19	0.7663	0.1699	−0.0142	0.092*	
C20	0.6910 (3)	0.1556 (3)	0.1697 (3)	0.0586 (7)	
H20	0.7135	0.0607	0.1954	0.070*	
C21	0.7259 (3)	−0.0736 (3)	0.8050 (3)	0.0553 (7)	
C22	0.7176 (3)	−0.2009 (3)	0.8816 (3)	0.0663 (8)	
H22	0.6435	−0.2339	0.8862	0.080*	
C23	0.8165 (4)	−0.2796 (4)	0.9508 (4)	0.0905 (12)	
H23	0.8101	−0.3658	1.0010	0.109*	
C24	0.9252 (4)	−0.2314 (5)	0.9463 (4)	0.1019 (14)	
H24	0.9920	−0.2843	0.9940	0.122*	
C25	0.9347 (4)	−0.1054 (5)	0.8716 (4)	0.0970 (13)	
H25	1.0080	−0.0723	0.8690	0.116*	
C26	0.8370 (3)	−0.0268 (4)	0.8002 (4)	0.0770 (10)	
H26	0.8456	0.0582	0.7484	0.092*	
C27	0.3063 (3)	0.2749 (3)	0.6210 (3)	0.0512 (7)	
C28	0.5913 (3)	0.1608 (3)	0.3930 (3)	0.0622 (8)	
H28A	0.4903	0.1859	0.4229	0.075*	
H28B	0.6220	0.0627	0.4037	0.075*	
C29	0.6160 (3)	0.0108 (3)	0.7288 (3)	0.0600 (8)	
H29A	0.6461	−0.0092	0.6481	0.072*	
H29B	0.5289	−0.0148	0.7704	0.072*	
C30	0.8121 (3)	0.1484 (4)	0.4424 (4)	0.0864 (12)	
H30A	0.8473	0.1766	0.4943	0.130*	
H30B	0.8494	0.1865	0.3563	0.130*	
H30C	0.8401	0.0505	0.4600	0.130*	
C31	0.5236 (4)	0.2029 (4)	0.8241 (3)	0.0922 (13)	
H31A	0.5110	0.2996	0.8052	0.138*	
H31B	0.5819	0.1564	0.8842	0.138*	
H31C	0.4340	0.1831	0.8575	0.138*	

### Atomic displacement parameters (Å^2^)

**Table:**

	*U*^11^	*U*^22^	*U*^33^	*U*^12^	*U*^13^	*U*^23^
P1	0.0399 (3)	0.0435 (4)	0.0562 (4)	−0.0113 (3)	−0.0114 (3)	−0.0136 (3)
N2	0.0407 (10)	0.0409 (12)	0.0630 (15)	−0.0116 (9)	−0.0131 (10)	−0.0100 (10)
N3	0.0524 (12)	0.0660 (16)	0.0568 (15)	−0.0061 (11)	−0.0139 (11)	−0.0230 (12)
N4	0.0536 (12)	0.0533 (14)	0.0517 (14)	−0.0087 (10)	−0.0142 (11)	−0.0167 (11)
O5	0.0465 (9)	0.0476 (11)	0.0894 (15)	−0.0148 (8)	−0.0206 (10)	−0.0161 (10)
O6	0.0572 (11)	0.0446 (12)	0.1014 (18)	−0.0157 (9)	−0.0223 (11)	−0.0092 (11)
F7	0.0756 (12)	0.0788 (14)	0.150 (2)	−0.0270 (10)	−0.0354 (13)	−0.0388 (14)
F8	0.0412 (9)	0.1202 (18)	0.1219 (19)	−0.0012 (10)	−0.0078 (10)	−0.0427 (15)
C9	0.0430 (13)	0.0523 (16)	0.0551 (17)	−0.0172 (12)	−0.0112 (12)	−0.0079 (13)
C10	0.0503 (15)	0.0680 (19)	0.065 (2)	−0.0196 (14)	−0.0077 (13)	−0.0238 (16)
C11	0.0461 (10)	0.0789 (16)	0.0738 (16)	−0.0124 (10)	−0.0027 (10)	−0.0261 (13)
C12	0.0461 (10)	0.0789 (16)	0.0738 (16)	−0.0124 (10)	−0.0027 (10)	−0.0261 (13)
C13	0.0479 (9)	0.0677 (13)	0.0672 (14)	−0.0239 (9)	−0.0129 (9)	−0.0101 (11)
C14	0.0479 (9)	0.0677 (13)	0.0672 (14)	−0.0239 (9)	−0.0129 (9)	−0.0101 (11)
C15	0.0556 (15)	0.0624 (18)	0.0538 (18)	−0.0246 (13)	−0.0113 (13)	−0.0154 (14)
C16	0.076 (2)	0.063 (2)	0.067 (2)	−0.0164 (16)	−0.0143 (17)	−0.0183 (17)
C17	0.088 (2)	0.077 (2)	0.070 (2)	−0.018 (2)	−0.022 (2)	0.002 (2)
C18	0.081 (2)	0.110 (3)	0.056 (2)	−0.025 (2)	−0.0169 (18)	−0.002 (2)
C19	0.0647 (19)	0.109 (3)	0.062 (2)	−0.0185 (19)	−0.0110 (16)	−0.036 (2)
C20	0.0537 (15)	0.071 (2)	0.0576 (19)	−0.0199 (14)	−0.0103 (14)	−0.0230 (16)
C21	0.0517 (15)	0.0591 (17)	0.0504 (17)	−0.0104 (13)	−0.0117 (13)	−0.0117 (14)
C22	0.0624 (17)	0.0610 (19)	0.064 (2)	−0.0105 (15)	−0.0121 (15)	−0.0088 (16)
C23	0.085 (3)	0.079 (3)	0.079 (3)	−0.004 (2)	−0.024 (2)	0.003 (2)
C24	0.072 (2)	0.113 (4)	0.095 (3)	0.013 (2)	−0.038 (2)	−0.014 (3)
C25	0.0576 (19)	0.119 (4)	0.112 (3)	−0.012 (2)	−0.036 (2)	−0.022 (3)
C26	0.0608 (18)	0.082 (2)	0.084 (3)	−0.0206 (17)	−0.0262 (17)	−0.005 (2)
C27	0.0479 (14)	0.0464 (16)	0.0629 (18)	−0.0154 (12)	−0.0173 (13)	−0.0107 (13)
C28	0.0791 (19)	0.0574 (18)	0.0570 (19)	−0.0301 (15)	−0.0025 (15)	−0.0216 (15)
C29	0.0641 (17)	0.0502 (17)	0.068 (2)	−0.0211 (14)	−0.0254 (15)	−0.0026 (15)
C30	0.0557 (18)	0.109 (3)	0.083 (3)	0.0049 (18)	−0.0099 (17)	−0.042 (2)
C31	0.098 (3)	0.105 (3)	0.059 (2)	0.006 (2)	−0.019 (2)	−0.034 (2)

### Geometric parameters (Å, °)

**Table:**

P1—O5	1.4787 (19)	C17—H17	0.9300
P1—N3	1.613 (2)	C18—C19	1.371 (5)
P1—N4	1.632 (2)	C18—H18	0.9300
P1—N2	1.683 (2)	C19—C20	1.386 (5)
N2—C27	1.370 (3)	C19—H19	0.9300
N2—H2	0.8600	C20—H20	0.9300
N3—C28	1.465 (4)	C21—C22	1.379 (4)
N3—C30	1.472 (4)	C21—C26	1.385 (4)
N4—C31	1.455 (4)	C21—C29	1.510 (4)
N4—C29	1.458 (4)	C22—C23	1.367 (5)
O6—C27	1.213 (3)	C22—H22	0.9300
F7—C14	1.351 (4)	C23—C24	1.372 (6)
F8—C12	1.357 (3)	C23—H23	0.9300
C9—C14	1.385 (4)	C24—C25	1.364 (6)
C9—C10	1.392 (4)	C24—H24	0.9300
C9—C27	1.487 (4)	C25—C26	1.375 (5)
C10—C11	1.381 (4)	C25—H25	0.9300
C10—H10	0.9300	C26—H26	0.9300
C11—C12	1.379 (5)	C28—H28A	0.9700
C11—H11	0.9300	C28—H28B	0.9700
C12—C13	1.345 (4)	C29—H29A	0.9700
C13—C14	1.370 (4)	C29—H29B	0.9700
C13—H13	0.9300	C30—H30A	0.9600
C15—C16	1.381 (4)	C30—H30B	0.9600
C15—C20	1.382 (4)	C30—H30C	0.9600
C15—C28	1.504 (4)	C31—H31A	0.9600
C16—C17	1.363 (5)	C31—H31B	0.9600
C16—H16	0.9300	C31—H31C	0.9600
C17—C18	1.373 (6)		
			
O5—P1—N3	114.10 (13)	C15—C20—C19	120.1 (3)
O5—P1—N4	110.83 (12)	C15—C20—H20	120.0
N3—P1—N4	108.04 (13)	C19—C20—H20	120.0
O5—P1—N2	106.78 (11)	C22—C21—C26	118.3 (3)
N3—P1—N2	108.02 (12)	C22—C21—C29	120.1 (3)
N4—P1—N2	108.93 (12)	C26—C21—C29	121.7 (3)
C27—N2—P1	125.86 (19)	C23—C22—C21	121.1 (3)
C27—N2—H2	117.1	C23—C22—H22	119.4
P1—N2—H2	117.1	C21—C22—H22	119.4
C28—N3—C30	114.7 (2)	C22—C23—C24	120.1 (4)
C28—N3—P1	127.0 (2)	C22—C23—H23	120.0
C30—N3—P1	118.1 (2)	C24—C23—H23	120.0
C31—N4—C29	114.2 (3)	C25—C24—C23	119.7 (4)
C31—N4—P1	117.2 (2)	C25—C24—H24	120.2
C29—N4—P1	125.2 (2)	C23—C24—H24	120.2
C14—C9—C10	116.4 (3)	C24—C25—C26	120.5 (4)
C14—C9—C27	121.6 (3)	C24—C25—H25	119.7
C10—C9—C27	122.0 (2)	C26—C25—H25	119.7
C11—C10—C9	121.9 (3)	C25—C26—C21	120.4 (4)
C11—C10—H10	119.1	C25—C26—H26	119.8
C9—C10—H10	119.1	C21—C26—H26	119.8
C12—C11—C10	117.2 (3)	O6—C27—N2	121.8 (2)
C12—C11—H11	121.4	O6—C27—C9	122.0 (2)
C10—C11—H11	121.4	N2—C27—C9	116.2 (2)
C13—C12—F8	118.3 (3)	N3—C28—C15	112.2 (2)
C13—C12—C11	124.0 (3)	N3—C28—H28A	109.2
F8—C12—C11	117.7 (3)	C15—C28—H28A	109.2
C12—C13—C14	117.0 (3)	N3—C28—H28B	109.2
C12—C13—H13	121.5	C15—C28—H28B	109.2
C14—C13—H13	121.5	H28A—C28—H28B	107.9
F7—C14—C13	117.0 (3)	N4—C29—C21	112.5 (2)
F7—C14—C9	119.4 (3)	N4—C29—H29A	109.1
C13—C14—C9	123.6 (3)	C21—C29—H29A	109.1
C16—C15—C20	118.9 (3)	N4—C29—H29B	109.1
C16—C15—C28	120.8 (3)	C21—C29—H29B	109.1
C20—C15—C28	120.3 (3)	H29A—C29—H29B	107.8
C17—C16—C15	120.8 (3)	N3—C30—H30A	109.5
C17—C16—H16	119.6	N3—C30—H30B	109.5
C15—C16—H16	119.6	H30A—C30—H30B	109.5
C16—C17—C18	120.4 (4)	N3—C30—H30C	109.5
C16—C17—H17	119.8	H30A—C30—H30C	109.5
C18—C17—H17	119.8	H30B—C30—H30C	109.5
C19—C18—C17	119.8 (4)	N4—C31—H31A	109.5
C19—C18—H18	120.1	N4—C31—H31B	109.5
C17—C18—H18	120.1	H31A—C31—H31B	109.5
C18—C19—C20	120.0 (3)	N4—C31—H31C	109.5
C18—C19—H19	120.0	H31A—C31—H31C	109.5
C20—C19—H19	120.0	H31B—C31—H31C	109.5
			
O5—P1—N2—C27	−152.1 (2)	C28—C15—C16—C17	−179.7 (3)
N3—P1—N2—C27	84.8 (3)	C15—C16—C17—C18	−0.1 (6)
N4—P1—N2—C27	−32.3 (3)	C16—C17—C18—C19	−0.2 (6)
O5—P1—N3—C28	−133.5 (2)	C17—C18—C19—C20	0.3 (6)
N4—P1—N3—C28	102.7 (3)	C16—C15—C20—C19	−0.1 (4)
N2—P1—N3—C28	−15.0 (3)	C28—C15—C20—C19	179.8 (3)
O5—P1—N3—C30	51.3 (3)	C18—C19—C20—C15	−0.2 (5)
N4—P1—N3—C30	−72.4 (3)	C26—C21—C22—C23	−0.2 (5)
N2—P1—N3—C30	169.9 (2)	C29—C21—C22—C23	179.6 (3)
O5—P1—N4—C31	55.7 (3)	C21—C22—C23—C24	1.0 (6)
N3—P1—N4—C31	−178.6 (2)	C22—C23—C24—C25	−0.7 (7)
N2—P1—N4—C31	−61.5 (3)	C23—C24—C25—C26	−0.5 (7)
O5—P1—N4—C29	−146.5 (2)	C24—C25—C26—C21	1.2 (6)
N3—P1—N4—C29	−20.8 (3)	C22—C21—C26—C25	−0.9 (5)
N2—P1—N4—C29	96.3 (2)	C29—C21—C26—C25	179.3 (3)
C14—C9—C10—C11	−0.7 (5)	P1—N2—C27—O6	−18.5 (4)
C27—C9—C10—C11	−179.1 (3)	P1—N2—C27—C9	159.9 (2)
C9—C10—C11—C12	−0.4 (5)	C14—C9—C27—O6	−34.0 (5)
C10—C11—C12—C13	1.2 (6)	C10—C9—C27—O6	144.4 (3)
C10—C11—C12—F8	−177.8 (3)	C14—C9—C27—N2	147.6 (3)
F8—C12—C13—C14	178.3 (3)	C10—C9—C27—N2	−34.1 (4)
C11—C12—C13—C14	−0.7 (5)	C30—N3—C28—C15	−66.2 (4)
C12—C13—C14—F7	−178.3 (3)	P1—N3—C28—C15	118.5 (3)
C12—C13—C14—C9	−0.6 (5)	C16—C15—C28—N3	−58.5 (4)
C10—C9—C14—F7	178.9 (3)	C20—C15—C28—N3	121.6 (3)
C27—C9—C14—F7	−2.7 (5)	C31—N4—C29—C21	−68.0 (3)
C10—C9—C14—C13	1.2 (5)	P1—N4—C29—C21	133.6 (2)
C27—C9—C14—C13	179.7 (3)	C22—C21—C29—N4	148.0 (3)
C20—C15—C16—C17	0.2 (5)	C26—C21—C29—N4	−32.2 (4)

### Hydrogen-bond geometry (Å, °)

**Table:**

*D*—H···*A*	*D*—H	H···*A*	*D*···*A*	*D*—H···*A*
N2—H2···O5^i^	0.86	1.96	2.812 (3)	171

Symmetry codes: (i) −*x*+1, −*y*+1, −*z*+1.
